# A Narrative Review of Current Status and Future Perspective of Telemedicine for Parkinson's Disease, Dementia, and Intractable Neurological Diseases in Japan

**DOI:** 10.14789/jmj.JMJ22-0031-R

**Published:** 2023-01-26

**Authors:** GENKO OYAMA, MAYUKO OGAWA, SATOKO SEKIMOTO, TAKU HATANO, NOBUTAKA HATTORI

**Affiliations:** 1 Department of Neurology, Juntendo University Graduate School of Medicine, Tokyo, Japan; 1 Department of Neurology, Juntendo University Graduate School of Medicine, Tokyo, Japan; 2 Department of Neurodegenerative and Demented Disorders, Juntendo University Graduate School of Medicine, Tokyo, Japan; 2 Department of Neurodegenerative and Demented Disorders, Juntendo University Graduate School of Medicine, Tokyo, Japan; 3 Department of Home Medical Care System based on Information and Communication Technology, Juntendo University Graduate School of Medicine, Tokyo, Japan; 3 Department of Home Medical Care System based on Information and Communication Technology, Juntendo University Graduate School of Medicine, Tokyo, Japan; 4 Department of Drug Development for Parkinson's Disease, Juntendo University Graduate School of Medicine, Tokyo, Japan; 4 Department of Drug Development for Parkinson's Disease, Juntendo University Graduate School of Medicine, Tokyo, Japan; 5 Department of PRO-Based Integrated Data Analysis in Neurological Disorders, Juntendo University Graduate School of Medicine, Tokyo, Japan; 5 Department of PRO-Based Integrated Data Analysis in Neurological Disorders, Juntendo University Graduate School of Medicine, Tokyo, Japan; 6 Department of Research and Therapeutics for Movement Disorders, Juntendo University Graduate School of Medicine, Tokyo, Japan; 6 Department of Research and Therapeutics for Movement Disorders, Juntendo University Graduate School of Medicine, Tokyo, Japan

**Keywords:** Parkinson's disease, dementia, telemedicine, wearable devices, artificial intelligence

## Abstract

The coronavirus disease 2019 pandemic has uncovered several inherent problems in society. While the demand for telemedicine surged worldwide and some countries responded flexibly, in Japan, most telemedicine services were limited to telephone consultations, and full-fledged telemedicine did not become widespread. In addition, the digitalization process in both medicine and wider society lags behind some other nations. It is necessary to accelerate digital transformation in healthcare to build a sustainable society that is resilient to crises, such as new pandemics. In particular, as Japan is facing an issue of super-aged society, a sustainable care model for people with Parkinson's disease, dementia, and intractable neurological diseases should be established.

Many neurodegenerative and intractable neurological diseases are progressive; as the disease progresses, patients could become difficult to visit specialists. Although online medical care has many advantages, it does not provide the same quality of information as face-to-face consultations.

However, new technology can overcome the limitations of online medical care. As an evolutionary direction for telemedicine, three-dimensional telemedicine technologies are being developed, which enable online medical treatment to be delivered as if the patient was sharing the same space. Telemonitoring can enable the objective and continuous evaluation of patient information at home through the use of motion capture, wearable devices, and other devices. The advancement of digital transformation in medical care should be a game-changer in accumulating big data and analyzing it using artificial intelligence.

## Introduction

The coronavirus disease 2019 (COVID-19) pandemic uncovered inherent problems in society. While the demand for telemedicine surged worldwide and some countries responded flexibly^1)^, in Japan, most telemedicine services were limited to telephone consultations, and full-fledged telemedicine did not become widespread^2)^. Moreover, the digitalization process in medicine and wider society lags behind some other nations. It is necessary to accelerate digital transformation (DX) in healthcare to build a sustainable society that is resilient to crises, such as new pandemics. In particular, as Japan is facing an issue of super-aged society, a sustainable care model for people with Parkinson's disease, dementia, and intractable neurological disorders should be established^3)^.

Many neurodegenerative and intractable neurological diseases are progressive, making patients increasingly difficult as the disease progresses to visit a specialist that could be located some distance away^4)^. In addition, symptoms may fluctuate within a day, and for appropriate management, it is necessary to accurately monitor not only symptoms during outpatient visits but also fluctuations in symptoms at home. Currently, however, few methods can objectively identify symptoms. Therefore, the importance of information and communication technology (ICT) applications and DX in healthcare is recognized as key to solving these problems.

Telemedicine technology can improve access to medical specialists. With the spread of COVID-19, telemedicine has gradually spread in Japan^2)^. Augmented reality (AR) and virtual reality (VR) technologies are expected to advance telemedicine technology and be applied to telerehabilitation and other areas. Wearable devices are a solution to objectively monitor patient conditions. By monitoring the patient 24 h a day with wearable devices, it is possible to objectively and continuously evaluate the patient's condition at home outside of medical examinations. In addition, the vast amount of big data obtained from monitoring with wearable devices may lead to the discovery of digital biomarkers and the development of diagnostic and therapeutic assistance programs through analysis using artificial intelligence (AI).

This review provides an overview of the current status and future perspectives of ICT and DX research in intractable neurological diseases.

## Methods

In this narrative review, we searched the literature on the current status of telemedicine in Parkinson's disease and intractable neurological diseases in Japan, published in English in PubMed. A search strategy identified relevant references using the terms Parkinson's disease, intractable neurological diseases, and telemedicine. Only original articles were included. We also discussed the limitation of current telemedicine and future perspectives of ICT and DX research to improve telemedicine.

## Results

We found that five articles fulfilled the criteria^2, 4)-7)^. Four studies include PD^2, 4, 6, 7)^, one study includes amyotrophic lateral sclerosis, spinocerebellar degeneration, and multiple system atrophy, as well as PD^6)^, and one study included spinocerebellar ataxia^5)^. Three research used tablet^2, 4, 7)^ and two research used telephone as ICT^5, 6)^.

## Discussion

### Telemedicine and online medical care

Many intractable neurological diseases are progressive, and as they progress, it becomes difficult for patients to visit specialists that may be located some distance away. In particular, aging is a risk factor for neurodegenerative diseases such as Parkinson's disease, and with the advent of an aging society, the number of intractable neurological diseases is increasing^8)^. However, because specialists are unevenly distributed in urban areas, it is difficult for patients with intractable neurological diseases in rural areas, where there are many elderly people, to access specialist care^4)^. One solution to improving access to specialists is telemedicine, which provides medical care remotely, and telehealth, which provides prevention and health promotion. Regardless of distance, health services using ICT are called eHealth or digital health and include various types of devices, such as wearable devices and smartphone applications^9)^.

In telemedicine, the Ministry of Health, Labour and Welfare (MHLW) in Japan defines “online medical care” as real-time medical treatment between a doctor and a patient, in which the doctor examines and diagnoses the patient using ICT devices and transmits diagnostic results and prescriptions^10)^. To date, there have been several restrictions on online medical care covered by national insurance. It is limited to patients with chronic disease and followed up for at least three months. Face-to-face visits must be scheduled at least every three months, with emergency cases seen within 30 min. Since the revision in 2022, fees for online medical care have also increased, similar to face-to-face consultations. In addition, time and distance requirements between medical institutions and patients, as well as limitations on the percentage of online medical care, were eliminated. Therefore, online medical care has been promoted.

### Telemedicine for Parkinson's disease, dementia, and intractable neurological disease

A previous review reported that telemedicine is an effective tool for the rapid evaluation of patients in remote locations that require neurological care for various neurological diseases, including dementia, neuromuscular diseases, multiple sclerosis, headache, trauma, and movement disorders^11)^. Since the first study reporting the feasibility of remote assessment of motor symptoms in Parkinson's disease in 1993^12)^, studies, including randomized controlled studies, have reported the usefulness of videoconferencing telemedicine in this field^4)^.

**Figure 1:**
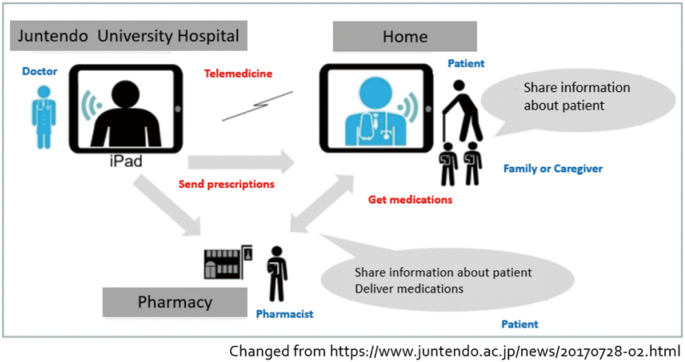
Telemedicine service in Juntendo University Hospital

In Japan, the first pilot study of telemedicine for Parkinson's disease using an iPad was conducted in 2014^4)^. This study showed the safety and feasibility of telemedicine care for patients with Parkinson's disease. Following this evidence, Juntendo University Hospital started a telemedicine service for Parkinson's disease and other intractable neurological diseases in 2017^2)^. In this service, the physician sees patients through the iPad and sends a prescription to patients as needed. Then, patients can get their medication from their nearest pharmacy (Figure 1). This service is now commonly provided in Japan. In a survey conducted during the first year of the service, both patients and caregivers were highly satisfied with the service and particularly appreciated the reduction in the burden of hospital visits^2)^. In 2018, the MHLW approved “online medical care” in telemedicine to be covered by national insurance. Although the number of users of our telemedicine service was limited to approximately 20 patients per month, the COVID- 19 pandemic led to a rapid five-fold increase in the number of users. We also started a trial of doctor-to-patient with doctor-type (D to P with D) online medical care and online second opinions. However, while demand for telemedicine surged and patients' tolerance for online medical care increased with the spread of COVID-19^13)^, most telemedicine services were limited to telephone consultations, and fully fledged telemedicine did not become widespread in Japan. In addition, there are limitations to the current online medical care services. Although online medical care has many advantages, it does not provide the same information as face-to-face consultations. The COVID-19 pandemic uncovered inherent problems in society and showed that medical care, as well as the whole society, is behind in the digitalization process. It is necessary to accelerate digital transformation in healthcare to build a sustainable society that is resilient to crises, such as new pandemics.

### Digital transformation for Parkinson's disease practice

Telemonitoring is a telemedicine technology that compensates for the weaknesses of current online medical technology. Using wearable devices and smartphone applications makes it possible to evaluate patient information objectively and continuously at home^14)^. Furthermore, an evolutionary direction for telemedicine includes technologies that enable three-dimensional video calls, facilitating online medical treatment as if the patients were sharing the same space as the doctors^15)^. Furthermore, digitization in medicine will allow the accumulation of big data and its analysis through AI.

### Three-dimensional telemedicine

One way to advance telemedicine treatment technology is the application of AR and mixed reality (MR) three-dimensional telemedicine technology. Three-dimensional telemedicine produces a three-dimensional hologram via a head-mounted display, which is scanned using an RGB depth camera in real-time^15)^. By wearing the head-mounted display, the doctor and patient can see each other and share the space as if they were right in front of each other, even though they are not. The advantages of three-dimensional online medical care are that it provides a more realistic examination environment and can be used to scan three-dimensional motion data of the entire body, which can be analyzed using AI. In the future, if AI-based algorithms can display relevant information in real-time in an MR space to assist physicians, such as frequency and the type of tremor, it might enhance the ability of non-specialists and provide an accurate evaluation aid.

**Figure 2:**
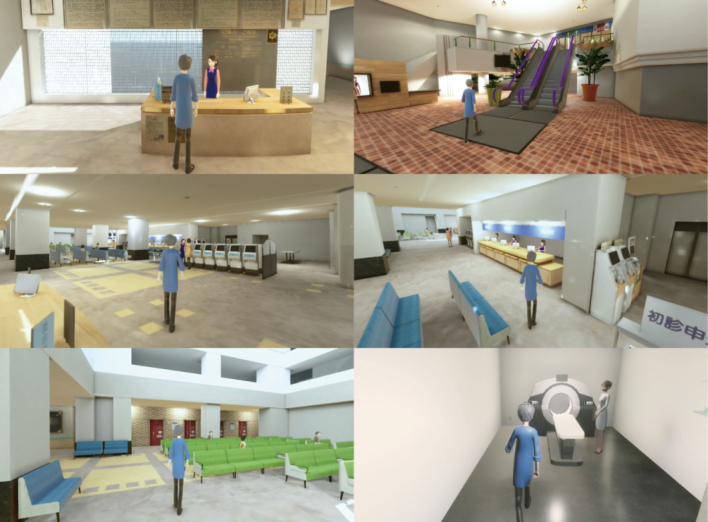
Virtual Juntendo hospital projects

In addition, medical applications of VR are underway^16)^. In particular, multiple studies on VR rehabilitation have been reported. The metaverse is the ultimate concept of further development of extended reality (XR) technologies, such as AR, VR, and MR. A metaverse is a three-dimensional virtual space or service built within a computer network. Users can freely experience various objects through their avatars in the metaverse space via computers or VR headsets. For example, at Juntendo University Hospital (Figure 2), a virtual hospital project has been started where patients and their families can virtually visit the hospital before coming, experience treatment methods, and interact with medical professionals and other patients (https://www.juntendo.ac.jp/news/20220413-05.html). It also allows hospitalized patients who have difficulty leaving the hospital to walk freely in a virtual space. In the future, we plan to examine whether mental health and other diseases can be improved through activities in the metaverse space.

### Wearable devices

In addition to XR technologies, another way to review symptoms that cannot be examined easily using telemedicine is the application of wearable devices. Wearable devices can continuously and objectively monitor changes in a patient's condition in daily life outside the hospital through 24 h continuous monitoring. Indeed, the Apple Watch offers a movement disorder API that can distinguish between tremors and dyskinesia and monitor them separately^17)^. Digital recording of an individual's movement symptoms is also expected to serve as a digital biomarker and is expected to become the basis for personalized medicine in the future^18)^.

### Artificial intelligence

AI is a computer program that imitates human intelligence. The basis of AI is machine learning, which includes supervised and unsupervised learning. The development of deep learning has dramatically improved the capabilities of AI. AI has already been used in medical applications in the field of diagnostic imaging, and research is underway to treat neurological disorders. For example, technology has been developed to automatically determine tremors by simply holding one's hand over an infrared motion capturing device^19)^.

We developed an AI-based chatbot application for telemedicine^7)^. Chatbots are programs that automatically converse with people, such as iPhone's Siri and Amazon's Alexa. Our AI-based chatbot was trained using several conversation scripts extracted from doctors' conversations with patients in their daily practice. Then, the AI-based chatbot selects from these scripts to converse with the patient. The application performs natural language processing based on the content of the conversation. In addition, it automatically displays a patient's health information report on a dashboard, which the doctor can use as a reference to provide regular or telemedicine care in less time. Our randomized controlled trial examined the efficacy and feasibility of the AI chatbot application. Twenty patients were randomized into a group that had only weekly remote conversations with the doctor and a group that had daily use of the AI chatbot in addition to a weekly conversation with their doctor to examine changes in facial expression and voice characteristics recorded during the remote discussion with physicians. AI chatbot intervention resulted in improvements in smiling and a decrease in filler words. This study indicated that daily conversations with an AI chatbot application could be applied to rehabilitation.

AI can save time for physicians by organizing the vast amount of information traffic, allowing physicians to focus on meaningful information, or by interviewing and rehabilitating patients instead of doctors and healthcare professionals. As a result, it might be possible for physicians to concentrate on their primary tasks, which only they can perform: listening to, sympathizing with, touching and examining, and healing patients.

### Limitations

The most significant limitation of this study is the paucity of studies. In addition, as this is a narrative review, no quality ratings of these references were performed. We need more crossectional and longitudinal studies to elucidate this field.

## Conclusion

**Figure 3:**
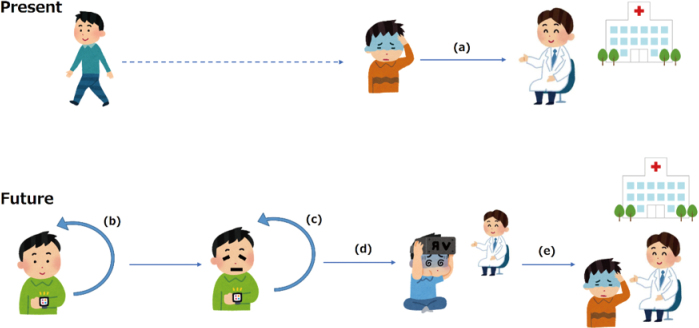
Paradigm shift in future medicine. (a) Patients see doctors when they get sick. (b)(c) People manage their health daily using wearable devices and smartphone apps. (d) People consult online when the devices highlight a problem. (e) Patients visit a hospital only when they need face-to-face medical care.

Various technologies, such as telemedicine, AR/VR/MR, metaverse, wearable devices, and AI technology, are being applied to treat intractable neurological diseases. The current limitations of telemedicine may be overcome by DX using new technologies, such as wearable devices, three-dimensional telemedicine, and AI. DX in medicine is expected to lead to a revolution in which various medical professionals can collaborate across the boundaries of professions and provide more effective medical care using fewer resources. In addition, a paradigm shift may occur in which instead of patients visiting a hospital after developing a disease, people manage their health daily using wearable devices and smartphone apps, consult online when the devices highlight a problem, and visit a hospital only when they need face-to-face medical care (Figure 3).

## Funding

This study was supported by a Grant-in-Aid from the Research Committee of CNS Degenerative Diseases, Research on Policy Planning and Evaluation for Rare and Intractable Diseases, Health, Labour, and Welfare Sciences Research Grants, and the Ministry of Health, Labour and Welfare, Japan (20FC1049), and a grant from the Japan Society for the Promotion of Science, Grants-in-Aid for Scientific Research (C) (#21K12711).

## Author contributions

The first draft of the manuscript was written by GO, and all authors commented on subsequent versions of the manuscript. All authors read and approved the final manuscript.

## Conflicts of interest statement

GO has received grants from Grant-in-Aid for Scientific Research (Kakenhi) and Novartis Pharma K.K. and honoraria from AbbVie Inc., Boston Scientific Corporation, Eisai Co., Ltd., Kyowa Kirin Co., Ltd., Medtronic Japan Co., Ltd., Otsuka Pharmaceutical Co., Ltd., Sumitomo Dainippon Pharma Co. Ltd., and Takeda Pharmaceutical Company Limited.

M.O. is an employee of Department of Neurodegenerative and Demented Disorders, Juntendo University Graduate School of Medicine, Tokyo, Japan.

SS declares that there are no conflicts of interest.

TH has received grants from: the Japan Agency for Medical Research and Development (grant numbers: 20dm0107156, 21wm0425015, 21ak0101112, and 21dk0207055), the Japan Society for the Promotion of Science, Grants-in-Aid for Scientific Research (grant number: 21K07424), the Setsuro Fujii Memorial Osaka Foundation for Promotion of Fundamental Medical Research, and Daiichi Sankyo TaNeDS; and research funds from: Daiichi Sankyo TaNeDS Funding Program. He has received speaker's honoraria from: Sumitomo Dainippon Pharma Co. Ltd., Takeda Pharmaceutical Company Limited, FP Pharmaceutical Corporation, Kyowa Kirin Co., Ltd., Nihon Medi-Physics Co., Ltd., Novartis Pharma K.K., Ono Pharmaceutical Co., Ltd, and Otsuka Pharmaceutical Co., Ltd.

NH has received consulting fees from GlaxoSmithKline K.K., AbbVie Inc., Eisai Co., Ltd., Otsuka Pharmaceutical Co., Ltd., Sumitomo Dainippon Pharma Co. Ltd., Kyowa Kirin Co., Ltd., Hisamitsu Pharmaceutical Co., Inc., Meiji Seika Pharma Co., Ltd., Ono Pharmaceutical Co., Ltd, and FP Pharmaceutical Corporation; lecture fees from MSD K.K., Eli Lilly Japan K.K., Eisai Co., Ltd., FP Pharmaceutical Corporation, Otsuka Pharmaceutical Co., Ltd., Tsumura & Co., Kyowa Kirin Co., Ltd., GlaxoSmithKline K.K., Takeda Pharmaceutical Company Limited, Mitsubishi Tanabe Pharma Corporation, Nihon Medi-Physics Co., Ltd., Novartis Pharma K.K., Pfizer Japan Inc., Nippon Boehringer Ingelheim Co., Ltd., Sumitomo Dainippon Pharma Co. Ltd., and Daiichi Sankyo Company, Limited; honoraria from FP Pharmaceutical Corporation, Novartis Pharma K.K., Kyowa Kirin Co., Ltd., and AbbVie Inc.; research support from Otsuka Pharmaceutical Co., Ltd.; and grants from Astellas Pharma Inc., Eisai Co., Ltd., GlaxoSmithKline K.K., Sumitomo Dainippon Pharma Co. Ltd., Takeda Pharmaceutical Company Limited, Novartis Pharma K.K., Pfizer Japan Inc., Kyowa Kirin Co., Ltd., Medtronic Japan Co., Ltd., Nippon Boehringer Ingelheim Co., Ltd., Boston Scientific Corporation, Kissei Pharmaceutical Co., Ltd, and Otsuka Pharmaceutical Co., Ltd.
